# Synthesis and structural characterization of a new dinuclear platinum(III) complex, [Pt_2_Cl_4_(NH_3_)_2_{μ-HN=C(O)Bu^t^}_2_]

**DOI:** 10.1107/S2052520622009660

**Published:** 2022-11-04

**Authors:** Doriana Vinci, Daniel Chateigner

**Affiliations:** a European XFEL, Holzkoppel 4, Schenefeld, 22869, Germany; b CRISMAT, CNRS, ENSICAEN, Université de Caen Normandie, Caen, France; Georgetown University, USA

**Keywords:** platinum, X-ray diffraction, crystal structure, antitumor drugs

## Abstract

Cisplatin plays an important role in treating many tumors. However, its pharmacological potential is limited by several side effects (nephrotoxicity, neurotoxicity, hair loss) and the resistance of many cancer types. In the effort of overcome resistance to cisplatin, more than 3000 platinum complexes have been synthesized in the past 45 years, but only about 30 have demonstrated adequate pharmacological platinum(III) complexes have been synthesized and their structure determined, because some of these exhibit antitumor and catalytic activity for the oxidation of olefins. Herein the synthesis of a new dinuclear platinum(III) complex with two butyl­(amidato) bridging ligands, namely [Pt_2_Cl_4_(NH_3_)_2_{μ-HN=C(O)Bu^*t*^}_2_], is reported. The complex was characterized by means of nuclear magnetic resonance (^1^H and ^195^Pt NMR) and infrared spectroscopy, and the crystal structure determined using X-ray single-crystal diffraction. This complex, to the best of our knowledge, is the first dinuclear complex of Pt^III^ with two butyl­(amidato) bridging ligands with a head-to-tail configuration.

## Abstract 2

The synthesis and the structural characterization of a new dinuclear platinum(III) complex are presented. Lantern-shaped platinum(III) complexes have been shown to have antitumor activity, so this new complex should be screened through antitumor screening tests.

## 1. Introduction

The interest in platinum complexes arises from their anti­cancer and catalytic activities for the oxidation of olefins. The discovery of the anticancer properties of cisplatin and its clinical introduction in the 1970s represent an important milestone in the history of effective anticancer drugs. Cisplatin, *cis*-diamminedi­chloro­platinum(II), was discovered by Rosenberg *et al.* (1965 ▸) during their experiments on the effect of electric fields on the cell division of bacteria. Cisplatin plays a significant role in the treatment of epithelial malignancies and in the cure of testicular cancer. It is used also as a principal component for the treatment of ovarian, head-and-neck, esophagus, stomach, colon, bladder, cervix and uterus cancers and as second-line treatment against most other advanced cancers including breast, pancreas, liver, kidney and prostate cancers. The primary cellular target of cisplatin is DNA, and the antitumor effects of platinum complexes are expected to arise from their ability to form several adducts with DNA, blocking its replication and transcription, and inducing cell death. The antitumor properties of cisplatin are attributed to the kinetics of the aqua­tion reactions of its chloride ligands leading to DNA crosslinking activities. This process is responsible of DNA bending, interferes with DNA replication, transcription and other nuclear functions, and blocks cancer cell proliferation and tumor growth (Boulikas *et al.*, 2007 ▸). However, its pharmacological potential is limited by its side effects (nephrotoxicity, neurotoxicity, hair loss), and the resistance of many cancer types (O’Dwyer *et al.*, 1999 ▸; Von Hoff *et al.*, 1979 ▸). In the past 45 years over 3000 platinum complexes have been synthesized in the effort of overcoming cisplatin limitations, but not more than 30 of them exhibited adequate pharmacological advantages over cisplatin. Only five of these latter complexes have been used in clinical applications, three FDA-approved (cisplatin, carboplatin and oxaliplatin), one used in Japan (nedaplatin) and one in China (lobaplatin). For a long time, the cisplatin geometry has been retained as a necessary requisite for a platinum complex to exhibit antitumoral activity (Kelland, 2007 ▸). Indeed, the *trans*-geometry of the platin complex and other transplatin analogs do not exhibit antitumor activity because of their kinetic instability which renders the complex unable to bind the target in the active form (Cornacchia *et al.*, 2009 ▸). More recently, however, several researchers have reported that the *trans*-isomer can gain cytotoxicity comparable to that of the *cis*-isomer and even of cisplatin (Farrell *et al.*, 1992 ▸; Natile & Coluccia, 2001 ▸; Montero *et al.*, 1999 ▸; Kasparkova *et al.*, 2003*a* ▸,*b* ▸).

**Figure 1:**
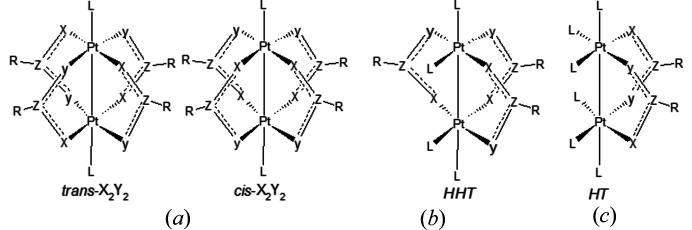
(*a*) ‘Lantern-type’ diplatinum(III) complex, (*b*) dinuclear Pt^III^ with three bridging ligands and (*c*) a ‘platinum blues’ derivate (this work). In this study, *YZX* = OCN, axial *L* = Cl, HHT is head-head-to-tail and HT is head-to-tail.

On the other hand, the number of dinuclear Pt^III^ complexes containing a metal–metal single bond is steadily increasing as well (O’Halloran & Lippard, 1985 ▸; Matsumoto & Sakai, 1999 ▸; González *et al.*, 2000 ▸; Saeki *et al.*, 2003 ▸). Dinuclear Pt^III^ complexes are interesting for their potential use in catalysis and biomedicine, and their chemistry and reactivity has not yet been explored to the same extent as for Pt^II^ and Pt^IV^ species. The majority of Pt^III^ complexes include a metal–metal single bond supported by two (Matsumoto & Sakai, 1999 ▸; Chen & Matsumoto, 2003 ▸; Lippert, 1999 ▸) or four (Fedotova *et al.*, 1997 ▸; Dolmella *et al.*, 2002 ▸; Bandoli *et al.*, 2003 ▸) bridging ligands (Fig. 1 ▸), namely ‘platinum blues’-derived and ‘lantern-type’, respectively. Dinuclear platinum complexes with three bridging ligands (Fig. 1 ▸) (Abe *et al.*, 1991 ▸) or unsupported by covalent bridges (Matsumoto *et al.*, 1996 ▸; Lippert *et al.*, 1983 ▸) are rare. The chelating chain usually consists of three atoms of the type O*X*O (*X* = C,S,P), NCO, NCS, SCS or P*X*P (*X* = C,O) resulting in an overall five-membered ring including the platinum–platinum interaction (Cornacchia *et al.*, 2009 ▸; Goodgame *et al.*, 1986 ▸; Umakoshi *et al.*, 1987 ▸; Cotton *et al.*, 1997 ▸; Bellitto *et al.*, 1983 ▸; Usón *et al.*, 1994 ▸). In addition to the bridging ligands, dinuclear Pt^III^ complexes also have equatorial and axial ligands (Matsumoto, 2003 ▸). A characteristic feature of dinuclear Pt^III^ complexes is an unusually long bond distance between the platinum and the axial ligands, which is 10% longer than the corresponding distances in square-planar Pt^II^ and octahedral Pt^IV^ coordinations (O’Dwyer *et al.*, 1999 ▸). Therefore, axial ligands are weaker bound, due to the strong *trans* labialization influence exerted by the intermetallic bond (Kuo *et al.*, 2007 ▸; Hartmann & Lipp, 2003 ▸; Dolmella *et al.*, 2002 ▸), and this characteristic feature may increase the reactivity toward the guanine bases of the DNA. The two-bridge Pt^III^ complexes are characterized by a tilting of the two platinum coordination planes of 25° and a twist about the platinum–platinum vector averaging a torsion angle of 25° (O’Halloran & Lippard, 1985 ▸). These distortions are due to the steric interactions between the non-bridging equatorial ligands.

In recent decades, the chemistry of dinuclear platinum complexes has attracted an increasing interest (O’Halloran & Lippard, 1985 ▸; Matsumoto & Sakai, 1999 ▸), because it has been demonstrated that some of these dinuclear Pt^III^ complexes exhibit antitumor (Cervantes *et al.*, 1997 ▸) and catalytic activities for the oxidation of olefins (Matsumoto & Sakai, 1999 ▸; Saeki *et al.*, 2003 ▸; Ochiai *et al.*, 2004 ▸). From this perspective, our research efforts have focused on the synthesis and the structural determination of a new head-to-tail (HT) dinuclear Pt^III^ complex (Fig. 1 ▸), with chemical formula [Pt_2_Cl_4_(NH_3_)_2_{μ-HN=C(O)Bu^*t*^}_2_]. The complex has been characterized by means of NMR and IR spectroscopies, while the structure was determined using X-ray single-crystal diffraction.

## 2. Experimental

### 2.1. Synthesis

(i) K_2_PtCl_4_ (2.1641 g, 5.21 mmol, *M*_r_ = 415.06 g mol^−1^) and KI (4.3874 g, 26.4 mmol, *M*_r_ = 165.99 g mol^−1^) were dissolved in water (15 ml). The solution was stirred at 55°C until a brown suspension consistent with the formation of the tetra­iodo salt, having formula K_2_PtI_4_, was observed. To this aqueous dispersion of potassium salt, an aqueous solution of NH_4_OH was added dropwise and the pH was controlled to not exceed 7.5 during the addition. The obtained solution was warmed at 55°C under stirring for 20 min to allow the formation of a yellow precipitate of *cis*-[PtI_2_(NH_3_)_2_]. 
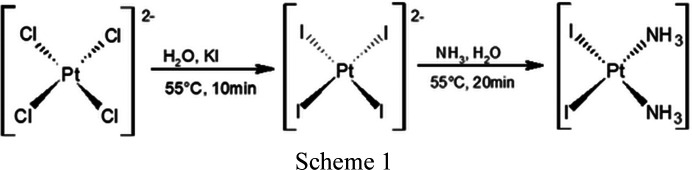


(ii) *cis*-[PtI_2_(NH_3_)_2_] (1.5229 g, 3.15 mmol, *M*_r_ = 482.92 g mol^−1^) was dissolved in water (20 ml), stirred with AgNO_3_ (1.0713 g, 6.31 mmol, *M*_r_ = 169.88 g mol^−1^) at 70°C for 20 min in the dark, and AgI was then removed by filtration from the yellowish suspension. The mother liquor was treated with NCBu^*t*^ (2 ml) and stirred at 70°C for 1 h, affording a blue solution of *cis*-[Pt(NH_3_)_2_(NCBu^*t*^)_2_]. The resulting nitrile complex was stirred at room temperature for 1 h with KI (2.6192 g, 17.78 mmol) to give a green precipitate of *trans*-[PtI_2_(NH_3_)(NCBu^*t*^)]. 
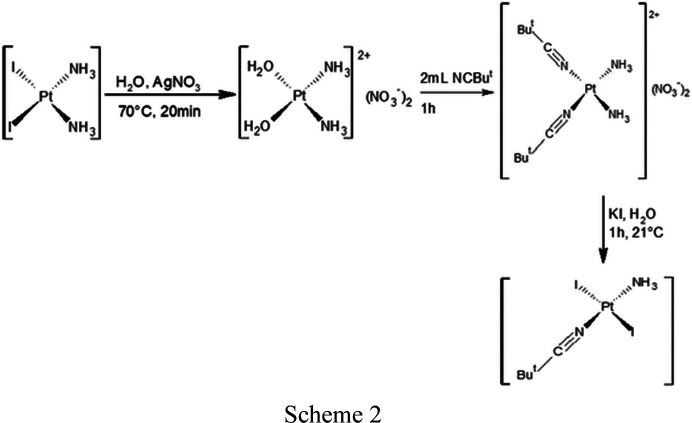


(iii) *trans*-[PtI_2_(NH_3_)(NCBu^*t*^)] (0.9457 g, 1.72 mmol, *M*_r_ = 549 g mol^−1^), dissolved in acetone (20 ml), was first stirred at 60°C to give a yellow solution, and then stirred with AgNO_3_ (0.5859 g, 3.45 mmol) at 70°C for 20 min in the dark, affording a green solution that was filtered. The mother solution was treated with KCl (1.2892 g, 17.3 mmol, *M*_r_ = 74.56 g mol^−1^) and then taken to dryness by evaporation of the solvent under reduced pressure. The solid residue was dissolved in water (20 ml), stirred at 50°C for 1 h, and dried in *vacuum* to obtain the formation of a green precipitate of *trans*-[PtCl_2_(NH_3_)(NCBu^*t*^)] . 
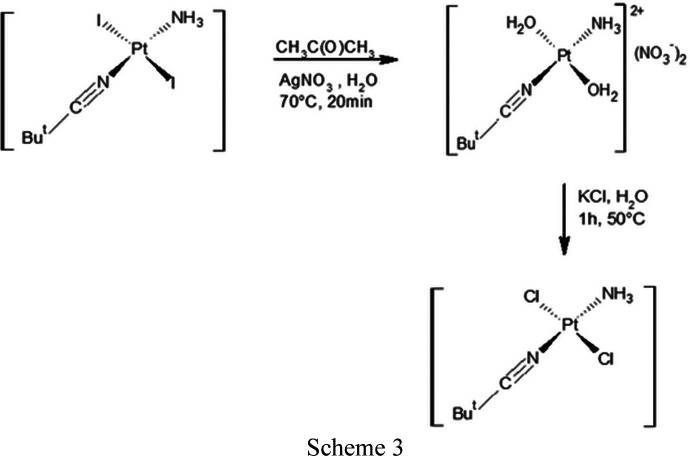


(iv) *trans*-[PtCl_2_(NH_3_)(NCBu^*t*^)] (0.1780 g, 0.49 mmol, *M*_r_ = 366 g mol^−1^) dissolved in chloro­form (30 ml) and Cl_2_ (2 ml) was stirred at room temperature for 30 min. The solvent was then evaporated under vacuum, forming a yellow precipitate of *trans*-[PtCl_4_(NH_3_)(NCBu^*t*^)]. 
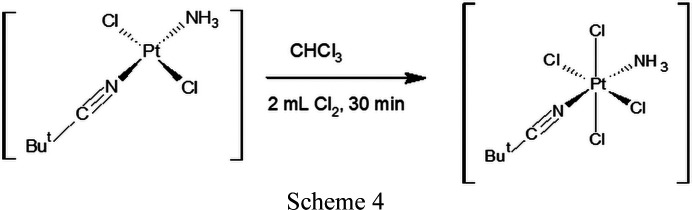


**Figure 2:**
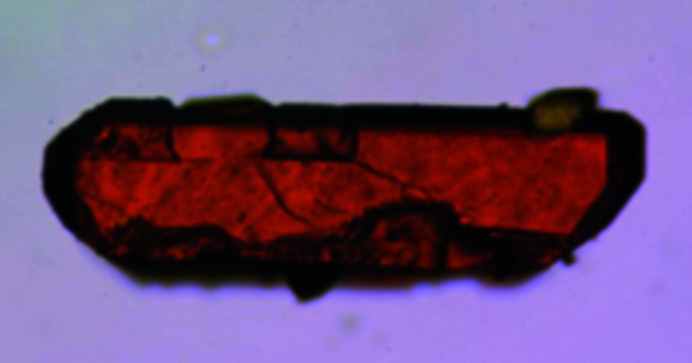
Single crystal selected for the X-ray diffraction characterization.

(v) The novel binuclear Pt^III^ complex can be prepared by the reaction of *trans*-[PtCl_4_(NH_3_)(NCBu^*t*^)] and *trans-*[PtI_2_(NH_3_)(NCBu^*t*^)] in water solution: *trans*-[PtCl_4_(NH_3_)(NCBu^*t*^)] (0.0335 g, 76.8 µmol, *M*_r_ = 436 g mol^−1^) and *trans*-[PtI_2_(NH_3_)(NCBu^*t*^)] (0.0415 g, 75.6 µmol) were first stirred at 60°C for 6 h in a water solution (10 ml), giving a brown suspension, and then heated for 20 h at 60°C. The solid residue was separated from the solution and crystallized in water by slow evaporation at room temperature. After three days, red crystals were formed (Fig. 2 ▸). It is worth noting that in the final complex only chlorine ligands are in the equatorial and axial positions. This can be explained by assuming that the iodide anion (from the Pt^II^ complex) is a stronger base with respect to chlorine, and as a consequence exhibits a larger reactivity with H_3_O^+^ cations (water after reacting with the platinum precursors releases hydrogen ions). 
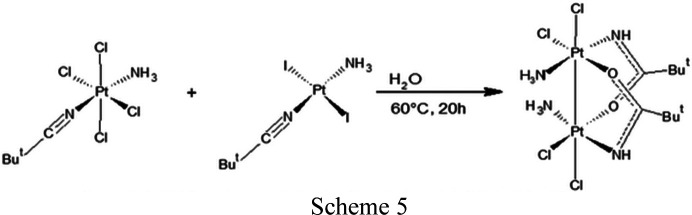


### 2.2. X-ray structure determination

The selected crystal (Fig. 1 ▸) was mounted on a Bruker AXS X8 APEX CCD diffractometer equipped with a four-circle Kappa goniometer and a 4 K CCD detector (radiation Mo *K*α). Data reduction and unit-cell refinement were carried out with the *SAINT* package (Bruker, 2003 ▸). A total of 29306 reflections (θ_max_ = 20.28°) was collected. The reflections were indexed, integrated and corrected for Lorentz, polarization and absorption effects with the program *SADABS* (Sheldrick, 2010 ▸). The unit-cell parameters were calculated from all reflections. The structure was solved using direct methods in space group *C*2/*c* and the model refined using full-matrix least-squares. The ADPs of non-hydrogen atoms were refined anisotropically, while hydrogen atoms were located by Fourier difference, except for those of the *tert*-butyl group which have been placed at calculated positions, and ADPs refined isotropically. All calculations and molecular graphics were carried out with *SIR92* (Altomare *et al.*, 1993 ▸), *PARST97* (Nardelli, 1995 ▸), *WinGX* (Ferrugia, 1999 ▸), *CRYSTALS* (Betteridge *et al.*, 2003 ▸), *MERCURY* (Macrae *et al.*, 2020 ▸) and *ORTEP-3* for Windows packages (Ferrugia, 2012 ▸). Details of the experiment and crystal data are listed in Table 1 ▸. Atomic positions are listed in Table 2 ▸. Selected bond lengths, bond angles and atomic displacement parameters are given in the CIF (see supporting information). The CIF file has been deposited in the Crystallography Open Database (Gražulis *et al.*, 2009 ▸; ref. 3000395).

## 3. Results and discussion

*NMR and IR characterization*. The ^1^H-NMR spectrum at 295 K in DMSO-*d*_6_ (Fig. S1) exhibits signals at frequencies ∼1.20, ∼5.00 and ∼6.70 ppm assigned to the *tert*-butyl [—C(CH_3_)_3_], ammine (—NH_3_) and amidate [—N(H)CO] protons, respectively. The [^1^H-^195^Pt] HSQC–NMR heterocorrelate spectrum (Fig. S2) recorded in DMSO-*d*_6_, exhibits two NH signals at 5.01 and 6.74 ppm correlated with the platinum signal at −347 ppm, indicative of a Pt^III^ cation in a N_2_Cl_2_OPt coordination environment. The occurrence of the *tert*-butyl and ammine ligands in the complex is coherent with the IR spectrum with bands at 2964–2918 and 3283–3405 cm^−1^, respectively (Fig. S2).

**Figure 3:**
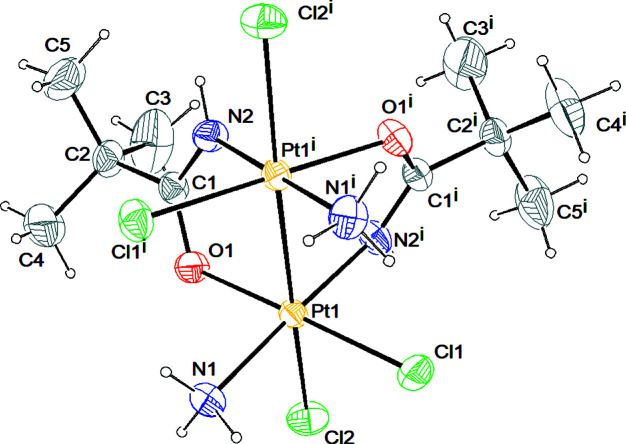
*ORTEP* drawing of the final structure model of the [Pt_2_Cl_4_(NH_3_)_2_{μ-HN=C(O)Bu^*t*^}] complex. Symmetry code: (i) 1 − *x*, *y*, ½ − *z*. Ellipsoids drawn at the 30% probability level. Atom color coding: white for hydrogen, green for chlorine, blue for nitrogen, gray for carbon and red for oxygen.

**Figure 4:**
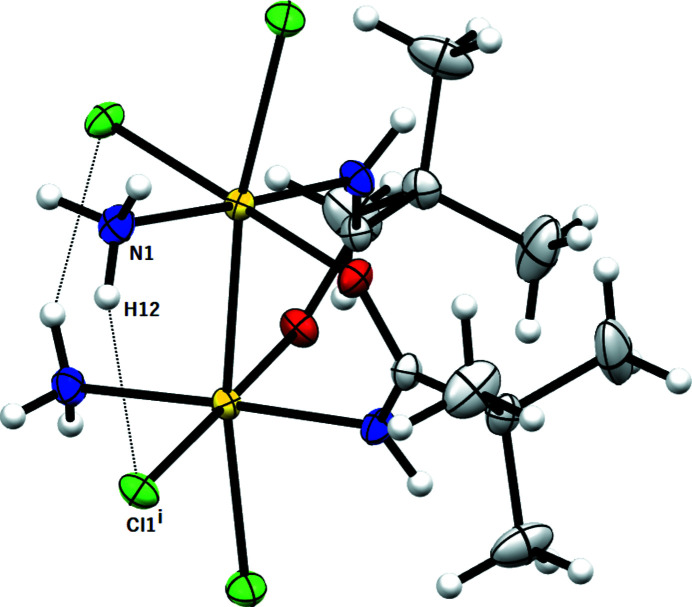
*MERCURY* drawing of [Pt_2_Cl_4_(NH_3_)_2_{μ-HN=C(O)Bu^*t*^}_2_] showing the intra­molecular hydrogen bond interaction [N1⋯Cl1^i^ 3.176 (3) Å, (N1)H12⋯Cl1^i^ 2.46 Å, N1—H12⋯Cl1^i^ 140°; symmetry code: (i) 1 − *x*, *y*, −*z* + ½]. Ellipsoids drawn at the 30% probability level. Atom color coding: white for hydrogen, green for chlorine, blue for nitrogen, gray for carbon and red for oxygen.

*X-ray diffraction analysis.* The asymmetric unit comprises half a molecule of the [Pt_2_Cl_4_(NH_3_)_2_{μ-HN=C(O)Bu^*t*^}_2_] complex and the structure is generated by the twofold axis at the midpoint of the Pt–Pt bond (Fig. 3 ▸). The coordination geometry of each Pt^III^ atom (Fig. 3 ▸) can be considered a distorted octahedron, with one chlorine, one oxygen and two nitro­gen atoms in equatorial positions, and one chlorine and one platinum of the second subunit in axial positions. The Pt–Pt bond distance [2.5661 (2) Å] is larger than that observed for four-bridge complexes with the same amidate bridge {*e.g.* [Pt_2_{HN=C(Bu^*t*^)O}_4_(9-EtG)_2_](NO_3_)_2_, 2.4512 (5) Å (Pacifico *et al.*, 2010 ▸)}, and in the range of ‘platinum blue’-derivates with the head-to-tail configuration [2.582 (1)–2.547 (1) Å range given by O’Halloran & Lippard (1985 ▸)]. This behavior indicates that the Pt–Pt distance is influenced by the number of bridging ligands. The equatorial Pt1—Cl1 [2.3214 (6) Å], Pt1—N1 [2.0576 (19) Å] and Pt—O1 [2.0243 (16) Å] bond distances are within the range of those reported for doubly and quadruply bridged dinuclear Pt^III^ (Fedotova *et al.*, 1997 ▸; Dolmella *et al.*, 2002 ▸; Hollis *et al.*, 1983 ▸), as well as for Pt^II^ and Pt^IV^ complexes (Erxleben *et al.*, 2002 ▸; Lippert *et al.*, 1984 ▸; Ali *et al.*, 2005 ▸; Sigel *et al.*, 1999 ▸; Shamsuddin *et al.*, 2007 ▸). Instead, the axial Pt1−Cl2 bond distance [2.4282 (6) Å] is longer than those reported in the literature (Fedotova *et al.*, 1997 ▸; Dolmella *et al.*, 2002 ▸; Hollis *et al.*, 1983 ▸; Erxleben *et al.*, 2002 ▸; Lippert *et al.*, 1984 ▸, 1986 ▸; Ali *et al.*, 2005 ▸; Sigel *et al.*, 1999 ▸; Shamsuddin *et al.*, 2007 ▸), possibly because of the strong *trans* influence exerted by the Pt—Pt bond. The doubly-bridged Pt^III^ molecule is characterized by a twist around the platinum–platinum vector [N2—Pt1—Pt1—O2] with an average torsion angle of 19.58°. The steric interactions between the ammine (bound to the first platinum) and the chloride (bound to the platinum in the second subunit) ligands are responsible for these distortions. In the amidate moiety, the C—*X* distances are 1.294 (3) Å and 1.293 (3) Å for the C1—N2 and C1—O1 bonds, respectively, in accordance with an extensive π-bond delocalization over the O—C—N moiety. The complex exhibits an intramolecular hydrogen bond involving the ammine hydrogen and the chlorine ligand (Fig. 4 ▸)

**Figure 5:**
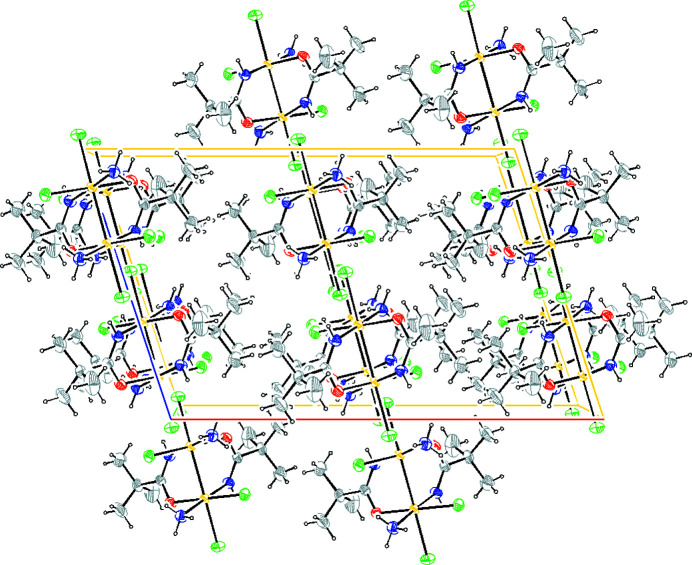
Crystal packing of [Pt_2_Cl_4_(NH_3_)_2_{μ-HN=C(O)Bu^*t*^}_2_], viewed along [010]. Ellipsoids drawn at the 30% probability level. Atom color coding: white for hydrogen, green for chlorine, blue for nitrogen, gray for carbon and red for oxygen.

**Figure 6:**
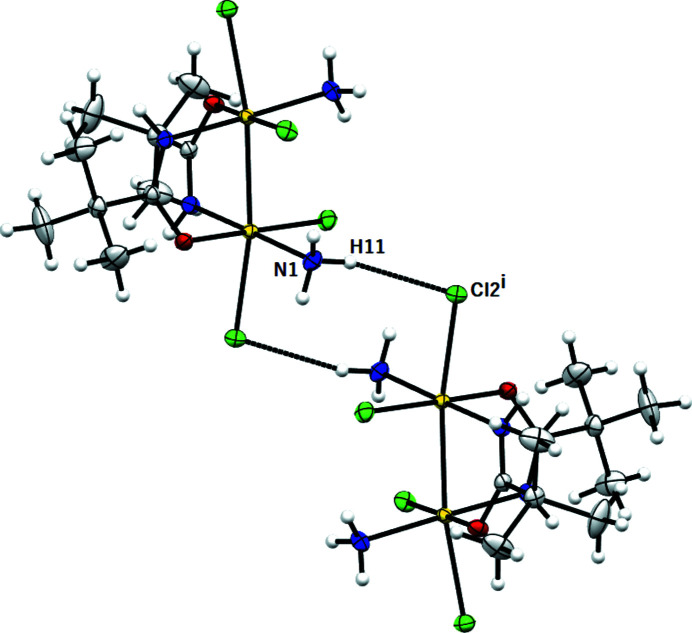
*MERCURY* drawing of two molecules of [Pt_2_Cl_4_(NH_3_)_2_{μ-HN=C(O)Bu^*t*^}_2_] linked by an intermolecular hydrogen bond [N1⋯Cl2^i^ 3.406 (2) Å, (N1)H11⋯Cl2^i^ 2.54 Å, N1—H1⋯Cl2^i^ 166°; symmetry code: (i) 1 − *x*, −*y*, 1 − *z*]. Ellipsoids drawn at the 30% probability level. Atom color coding: white for hydrogen, green for chlorine, blue for nitrogen, gray for carbon and red for oxygen.

**Figure 7:**
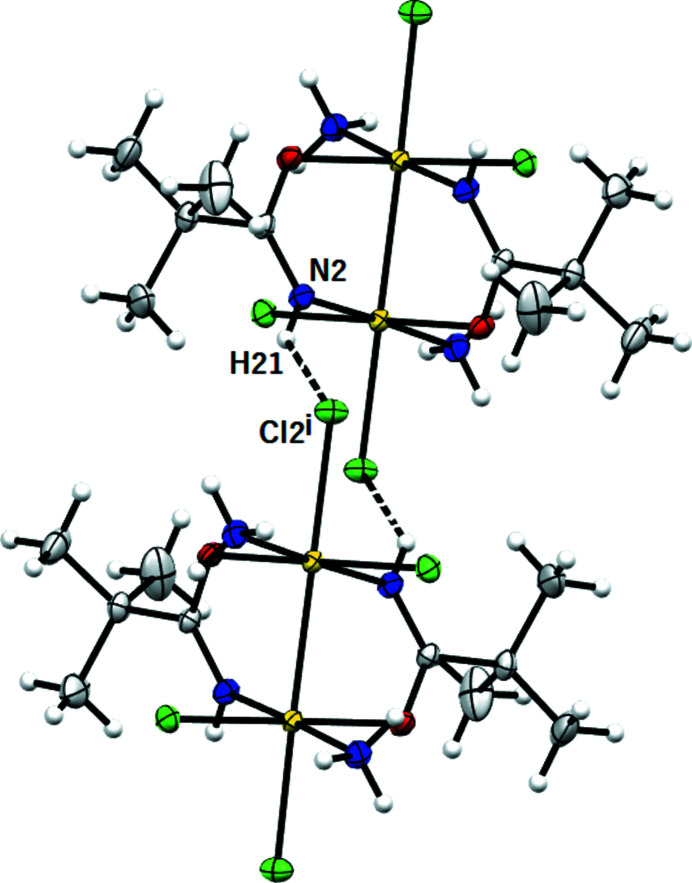
*MERCURY* drawing of two molecules of [Pt_2_Cl_4_(NH_3_)_2_{μ-HN=C(O)Bu^*t*^}_2_] linked by an intermolecular hydrogen bond [N2⋯Cl2^i^ 3.361 (2) Å, (N2)H21⋯Cl2^i^ 2.67 Å, N2—H21⋯Cl2^i^ 138°; symmetry code: (i) *x*, 1 − *y*, *z* + ½]. Ellipsoids drawn at the 30% probability level. Atom color coding: white for hydrogen, green for chlorine, blue for nitrogen, gray for carbon and red for oxygen.

**Figure 8:**
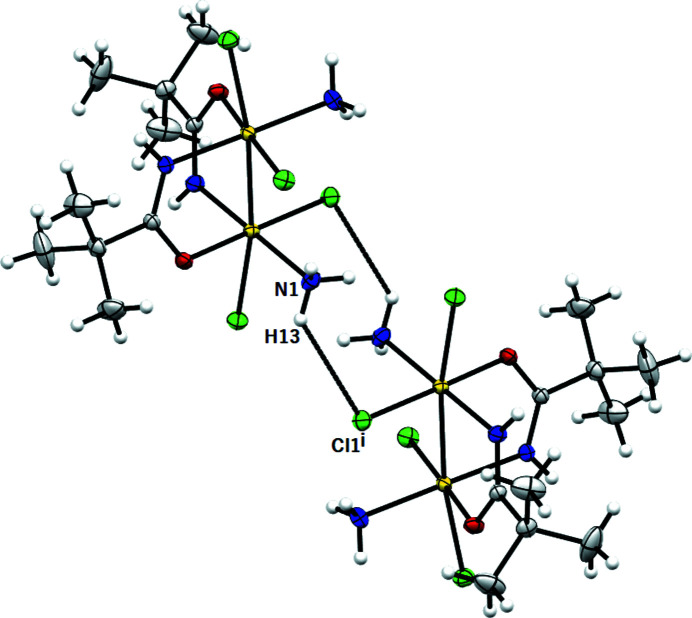
*MERCURY* drawing of two molecules of [Pt_2_Cl_4_(NH_3_)_2_{μ-HN=C(O)Bu^*t*^}_2_] linked by van der Waals intermolecular interactions [N1⋯Cl1^i^ 3.496 (2) Å, (N1)H13⋯Cl1^i^ 2.86 Å, N1—H13⋯Cl1^i^ 130°; symmetry code: (i) 1 − *x*, −*y*, 1 − *z*]. Ellipsoids drawn at the 30% probability level. Atom color coding: white for hydrogen, green for chlorine, blue for nitrogen, gray for carbon and red for oxygen.

The molecular packing (Fig. 5 ▸) is governed by intermolecular hydrogen bonds (Table 3 ▸) and van der Waals interactions. (i) Intermolecular hydrogen bonds occur between the N(H) hydrogen of the ammine group and the chlorine ligand (see Fig. 6 ▸); and between the axial chlorine and the N(H) hydrogen of the amidate chelating group (see Fig. 7 ▸). (ii) van der Waals intermolecular forces involve chlorine and ammine ligands in equatorial position (see Fig. 8 ▸).

The structural results from the spectroscopic and diffraction analyses confirm that we have successfully designed the synthesis of the expected complex, and the absence of iodine ligands as hypothesized.

## 4. Conclusion

The necessity to overcome the limitations associated with the use of cisplatin, such as the occurrence of toxic side effects and drug-resistance behaviors, has led to the synthesis of new complexes with higher pharmacological properties. In this study, we have identified a strategy for synthesizing a novel dinuclear Pt^III^ complex from the pivalo­nitrile derivatives of Pt^II^ and Pt^IV^ as precursors. The complex has been characterized by NMR (^1^H and ^195^Pt) and IR spectroscopies. The crystal structure was determined by X-ray crystallography. To our knowledge this complex is the first example of dinuclear Pt^III^ species with two bridging ligands in HT configuration. Since it has been demonstrated that ‘lantern-shaped’ Pt^III^ complexes exhibit antitumor activity, it will be interesting to investigate the activity of our complex with biological assays.

## Supplementary Material

Crystal structure: contains datablock(s) I. DOI: 10.1107/S2052520622009660/yv5006sup1.cif

Structure factors: contains datablock(s) 1. DOI: 10.1107/S2052520622009660/yv5006Isup2.hkl

Figs. S1, S2 and S3. DOI: 10.1107/S2052520622009660/yv5006sup3.pdf

CCDC reference: 2210784

## Figures and Tables

**Table 1:** Experimental details

Crystal data	
Chemical formula	[Pt_2_Cl_4_(NH_3_)_2_{μ-HN=C(O)Bu^*t*^}_2_]
*M*_r_	766.33
Crystal system, space group	Monoclinic, *C*2/*c*
Temperature (K)	293
*a*, *b*, *c* (Å)	19.3247 (3), 8.8795 (1), 12.7062 (2)
β (°)	108.332 (1)
*V* (Å^3^)	2069.65 (5)
*Z*	4
Radiation type	Mo *K*α
μ (mm^−1^)	14.03
Crystal size (mm)	0.77 × 0.24 × 0.21

Data collection	
Diffractometer	Nonius KappaCCD
Absorption correction	Multi-scan (*SADABS*)
*T*_min_, *T*_max_	0.03, 0.05
No. of measured, independent and observed [*I* > 2.0σ(*I*)] reflections	29306, 6496, 4944
*R*_int_	0.027
(sin θ/λ)_max_ (Å^−1^)	0.910

Refinement	
*R*[*F* ^2^ > 2σ(*F* ^2^)], *wR*(*F* ^2^), *S*	0.022, 0.017, 1.12
No. of reflections	4707
No. of parameters	100
H-atom treatment	H-atom parameters constrained
Δρ_max_, Δρ_min_ (e Å^−3^)	1.30, −1.30

Computer programs: *COLLECT* (Nonius, 2001 ▸), *CrysAlis* (Oxford Diffraction, 2002 ▸), *SIR92* (Altomare *et al.*, 1993 ▸), *CRYSTALS* (Betteridge *et al.*, 2003 ▸), *CAMERON* (Watkin *et al.*, 1996 ▸).

**Table 2:** Selected geometric parameters (Å, °)

Pt1—Pt1^i^	2.5661 (2)	Pt1—N2^i^	1.9989 (18)
Pt1—Cl1	2.3214 (6)	Pt1—O1	2.0243 (16)
Pt1—Cl2	2.4282 (6)	C1—O1	1.293 (3)
Pt1—N1	2.0576 (19)	C1—N2	1.294 (3)
Pt1^i^—Pt1—Cl2	170.852 (16)	Cl1—Pt1—N1	90.92 (6)
Pt1^i^—Pt1—O1	85.94 (5)	N2^i^—Pt1—O1	93.18 (8)
Pt1^i^—Pt1—N1	99.37 (6)	N2^i^—Pt1—Cl2	90.82 (6)
O1—C1—N2	121.65 (19)	N2^i^—Pt1—Cl1	87.80 (6)
Cl2—Pt1—O1	88.46 (5)	N2^i^—Pt1—N1	177.99 (9)
Cl2—Pt1—N1	87.66 (7)	N2^i^—Pt1—Pt1^i^	82.28 (6)
Cl1—Pt1—O1	179.02 (5)		

Symmetry code: (i) 1 − *x*, *y*, −*z* + ½.

**Table 3:** Hydrogen-bond geometry (Å, °)

*D*—H⋯A	*D*—H	H⋯A	*D*⋯*A*	*D*—H⋯*A*
Intramolecular				
N1—H11⋯Cl2^i^	0.89	2.464	3.176 (3)	140
Intermolecular				
N1—H11⋯Cl2^ii^	0.89	2.541	3.406 (2)	166
N2—H21⋯Cl2^iii^	0.87	2.669	3.361 (2)	138

Symmetry codes: (i) 1 + *x*, *y*, −*z* + ½; (ii) 1 − *x*, −*y*, 1 − *z*; (iii) *x*, 1 − *y*, *z* + ½.

## References

[bb1] Abe, T., Moriyama, H. & Matsumoto, K. (1991). *Inorg. Chem.* **30**, 4198–4204.

[bb2] Ali, M. S., Ali Khan, S. R., Ojima, H., Guzman, I. Y., Whitmire, K. H., Siddik, Z. H. & Khokhar, A. R. (2005). *J. Inorg. Biochem.* **99**, 795–804. 10.1016/j.jinorgbio.2004.12.015 15708801

[bb3] Altomare, A., Cascarano, G., Giacovazzo, C., Guagliardi, A., Camalli, M., Burla, M. C. & Polidori, G. (1993). *Acta Cryst.* A**49**, *c*55.

[bb4] Bandoli, G., Dolmella, A., Intini, F. P., Pacifico, C. & Intini, G. (2003). Inorg. Chim. Acta, **346**, 143–150.

[bb5] Bellitto, C., Flamini, A., Gastaldi, L. & Scaramuzza, L. (1983). *Inorg. Chem.* **22**, 444–449.

[bb6] Betteridge, P. W., Carruthers, J. R., Cooper, R. I., Prout, K. & Watkin, D. J. (2003). *J. Appl. Cryst.* **36**, 1487–1487.

[bb7] Boulikas, T., Pantos, A., Bellis, E. & Christofis, P. (2007). *Cancer Ther.* **5**, 537–583.

[bb8] Bruker (2003). *SAINT*. Bruker AXS Inc., Madison, Wisconsin, USA.

[bb9] Cervantes, G., Prieto, M. J. & Moreno, V. (1997). *Met.-Based Drugs*, **4**, 9–18. 10.1155/MBD.1997.9 PMC2365033 18475760

[bb10] Chen, W. & Matsumoto, K. (2003). *Inorg. Chim. Acta*, **342**, 88–96.

[bb11] Cornacchia, D., Pellicani, R. Z., Intini, F. P., Pacifico, C. & Natile, G. (2009). *Inorg. Chem.* **48**, 10800–10810. 10.1021/ic9011372 19821556

[bb12] Cotton, F. A., Matonic, J. H. & Murillo, C. A. (1997). *Inorg. Chim. Acta*, **264**, 61–65.

[bb13] Dolmella, A., Intini, F. P., Pacifico, C., Padovano, G. & Natile, G. (2002). *Polyhedron*, **21**, 275–280.

[bb14] Erxleben, A., Metzger, S., Britten, J. F., Lock, C. J. L., Albinati, A. & Lippert, B. (2002). *Inorg. Chim. Acta*, **339**, 461–469.

[bb15] Farrell, N., Kelland, L. R., Roberts, J. D. & Van Beusichem, M. (1992). *Cancer Res.* **52**, 5065–5072. 1516063

[bb16] Fedotova, T. N., Minacheva, L. K., Kuznetsova, G. N., Sakharova, V. G., Gelfman, M. I. & Baranovskii, I. B. (1997). *Russ. J. Inorg. Chem.* **42**, 1838–1846.

[bb17] Ferrugia, L. J. (1999). *J. Appl. Cryst.* **32**, 837–838.

[bb18] Ferrugia, L. J. (2012). *J. Appl. Cryst.* **45**, 849–854.

[bb19] González, V. M., Fuertes, M. A., Pérez-Alvarez, M. J., Cervantes, G., Moreno, V., Alonso, C. & Pérez, J. M. (2000). *Biochem. Pharmacol.* **60**, 371–379. 10.1016/s0006-2952(00)00329-4 10856432

[bb20] Goodgame, D. M. L., Rollins, R. W., Slawin, A. M. Z., Williams, D. J. & Zard, P. W. (1986). *Inorg. Chim. Acta*, **120**, 91–101.

[bb21] Gražulis, S., Chateigner, D., Downs, R. T., Yokochi, A. F. T., Quirós, M., Lutterotti, L., Manakova, E., Butkus, J., Moeck, P. & Le Bail, A. (2009). *J. Appl. Cryst.* **42**, 726–729. 10.1107/S0021889809016690 PMC3253730 22477773

[bb22] Hartmann, J. T. & Lipp, H. P. (2003). *Expert Opin. Pharmacother.* **4**, 889–901. 10.1517/14656566.4.6.889 12783586

[bb23] Hollis, L. S., Roberts, M. M. & Lippard, S. J. (1983). *Inorg. Chem.* **22**, 3637–3644.

[bb24] Kasparkova, J., Marini, V., Najajreh, Y., Gibson, D. & Brabec, V. (2003*a*). *Biochemistry*, **42**, 6321–6332. 10.1021/bi0342315 12755637

[bb25] Kasparkova, J., Novakova, O., Farrell, N. & Brabec, V. (2003*b*). *Biochemistry*, **42**, 792–800. 10.1021/bi026614t 12534292

[bb26] Kelland, L. R. (2007). *Nat. Rev. Cancer*, **7**, 573–584. 10.1038/nrc2167 17625587

[bb27] Kuo, M. T., Chen, H. H., Song, I. S., Savaraj, N. & Ishikawa, T. (2007). *Cancer Metastasis Rev.* **26**, 71–83. 10.1007/s10555-007-9045-3 17318448

[bb28] Lippert, B. (1999). *Coord. Chem. Rev.* **182**, 263–295.

[bb29] Lippert, B., Raudaschl, G., Lock, C. J. L. & Pilon, P. (1984). *Inorg. Chim. Acta*, **93**, 43–50.

[bb30] Lippert, B., Schoellhorn, H. & Thewalt, U. (1986). *Inorg. Chem.* **25**, 407–408. 10.1021/ja00263a038 22175483

[bb31] Lippert, B., Schollhorn, H. & Thewalt, U. (1983). *Z. Naturforsch.* **38**, 1441–1445.

[bb32] Macrae, C. F., Sovago, I., Cottrell, S. J., Galek, P. T. A., McCabe, P., Pidcock, E., Platings, M., Shields, G. P., Stevens, J. S., Towler, M. & Wood, P. A. (2020). *J. Appl. Cryst.* **53**, 226–235. 10.1107/S1600576719014092 PMC6998782 32047413

[bb33] Matsumoto, K. (2003). *Russ. Chem. Bull.* **52**, 2577–2587.

[bb34] Matsumoto, K., Matsunami, J., Mizuno, K. & Uemura, H. (1996). *J. Am. Chem. Soc.* **118**, 8959–8960.

[bb35] Matsumoto, K. & Sakai, K. (1999). *Adv. Inorg. Chem.* **49**, 375–427.

[bb36] Montero, E. I., Díaz, S., González-Vadillo, A. M., Pérez, J. M., Alonso, C. & Navarro-Ranninger, C. (1999). *J. Med. Chem.* **42**, 4264–4268. 10.1021/jm991015e 10514297

[bb37] Nardelli, M. (1995). *J. Appl. Cryst.* **28**, 659–659.

[bb38] Natile, G. & Coluccia, M. (2001). *Coord. Chem. Rev.* **216**–**217**, 383–410.

[bb100] Nonius (2001). *COLLECT*. Nonius BV, Delft, The Netherlands.

[bb39] Ochiai, M., Lin, Y.-S., Yamada, J., Misawa, H., Arai, S. & Matsumoto, K. (2004). *J. Am. Chem. Soc.* **126**, 2536–2545. 10.1021/ja0302634 14982463

[bb40] O’Dwyer, P., Stevenson, J. & Johnson, S. (1999). In *Cisplatin: Chemistry and Biochemistry of a Leading Anticancer Drug*, edited by B. Lippert. Zurich: Verlag Helvetica Chimica Acta.

[bb41] O’Halloran, T. V. & Lippard, S. J. (1985). *Isr. J. Chem.* **25**, 130–137.

[bb102] Oxford Diffraction (2002). *CrysAlis*. Oxford Diffraction Ltd, Abingdon, Oxfordshire, England.

[bb42] Pacifico, C., Intini, F. P., Nushi, F. & Natile, G. (2010). *Bioinorg. Chem. Appl.* **2010**, 102863. 10.1155/2010/102863 PMC2901605 20631827

[bb43] Rosenberg, B., Van Camp, L. & Krigas, T. (1965). *Nature*, **205**, 698–699. 10.1038/205698a0 14287410

[bb44] Saeki, N., Nakamura, N., Ishibashi, T., Arime, M., Sekiya, H., Ishihara, K. & Matsumoto, K. (2003). *J. Am. Chem. Soc.* **125**, 3605–3616. 10.1021/ja020953s 12643723

[bb45] Shamsuddin, S., Ali, M. S., Whitmire, K. H. & Khokhar, A. R. (2007). *Polyhedron*, **26**, 637–644.

[bb46] Sheldrick, G. M. (2010). *SADABS*. University of Gottingen, Gottingen, Germany.

[bb47] Sigel, R. K. O., Sabat, M., Freisinger, E., Mower, A. & Lippert, B. (1999). *Inorg. Chem.* **38**, 1481–1490.

[bb48] Umakoshi, K., Kinoshita, I., Ichimura, A. & Ooi, S. (1987). *Inorg. Chem.* **26**, 3551–3556.

[bb49] Usón, R., Forniés, J., Falvello, L. R., Tomas, M., Casas, J. M., Martin, A. & Cotton, F. A. (1994). *J. Am. Chem. Soc.* **116**, 7160–7165.

[bb50] Von Hoff, D. D., Schilsky, R., Reichert, C. M., Reddick, R. L., Rozencweig, M., Young, R. C. & Muggia, F. M. (1979). *Cancer Treat. Rep.* **63**, 1527–1531. 387223

[bb104] Watkin, D. J., Prout, C. K. & Pearce, L. J. (1996). *CAMERON*. Chemical Crystallography Laboratory, Oxford, England.

