# Supplementary material for: Synthesis and structural characterization of a new dinuclear platinum(III) complex, [Pt2Cl4(NH3)2{μ-HN=C(O)But}2]
Source: Acta Crystallogr B Struct Sci Cryst Eng Mater. 2022 Nov 4;78(Pt 6):835–41. doi: 10.1107/S2052520622009660 (PMC9728019; doi:10.1107/S2052520622009660)
Supplement: Supplementary file 3 [file b-78-00835-sup3.pdf]

STRUCTURAL SCIENCE
CRYSTAL ENGINEERING
MATERIALS

**Volume 78 (2022)**

**Supporting information for article:**

# Synthesis and structural characterization of a new dinuclear platinum(III) complex, [Pt2Cl4(NH3)2{μ-HN&z-dbnd;C(O)Bu$^{t}$}2]

**Doriana Vinci and Daniel Chateigner**

# Supporting materials

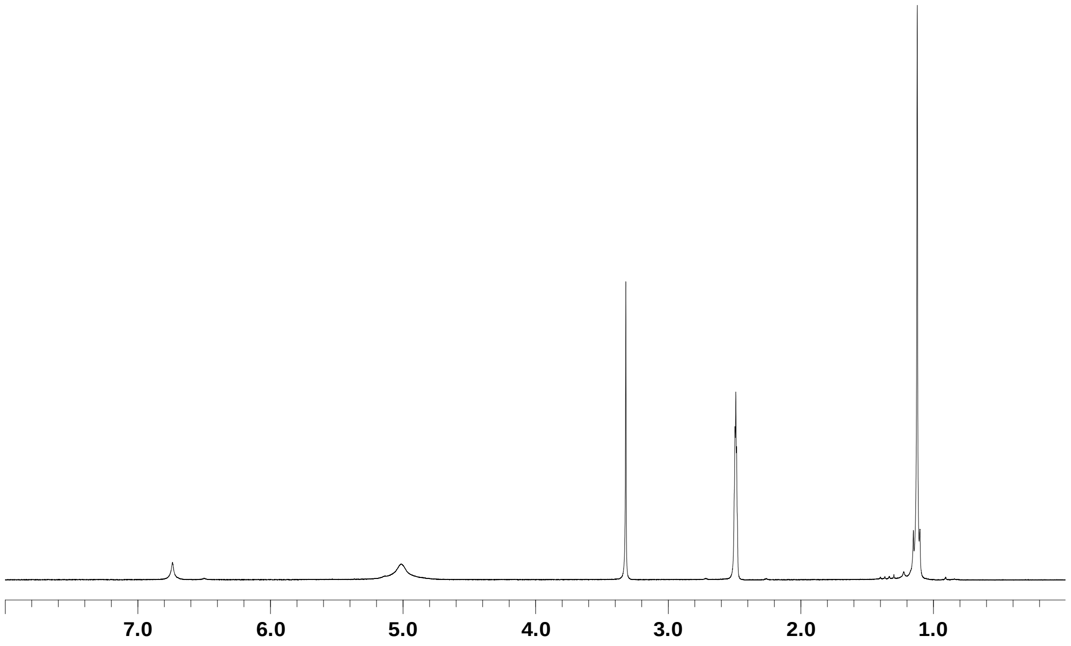

Figure S1. $^{1}$H-NMR spectrum at 295 K in DMSO-d$_6$. Signals at frequencies ~1.20, ~5.00, and ~6.70 ppm assigned to the *tert*-butyl (-C(CH$_3$)$_3$), ammine (-NH$_3$), and amidate (-N(H)CO)

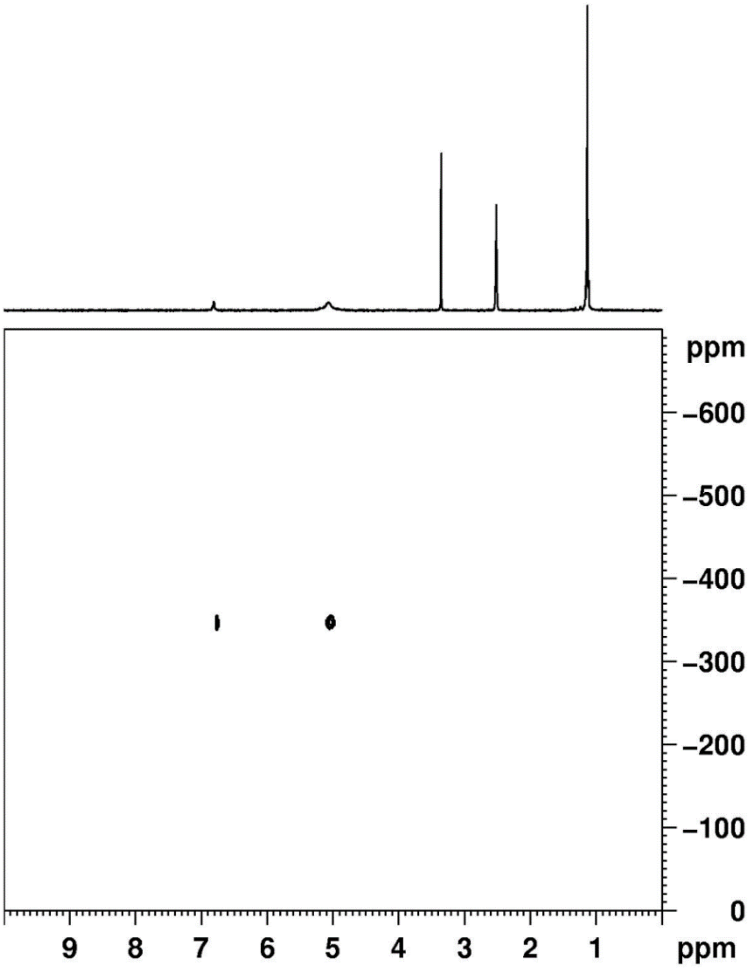


protons, respectively.

Figure S2. [$^{1}$H-$^{195}$Pt] HSQC-NMR heterocorrelate spectrum recorded in DMSO-d$_6$. The spectrum shows two NH signals at 5.01 and 6.74 ppm correlated with the platinum signal at -347 ppm, indicative of a Pt$^{III}$ cation in a $N_2Cl_2OPt$ coordination environment.

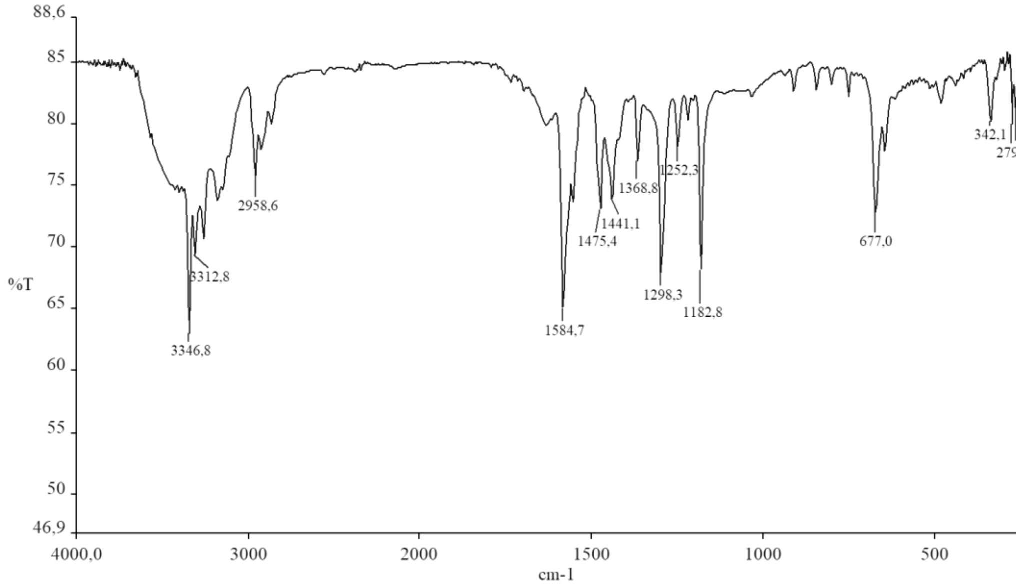


Figure S3. Infrared spectrum.
